# Development of an algorithm to identify serious opioid toxicity in children

**DOI:** 10.1186/s13104-015-1185-x

**Published:** 2015-07-04

**Authors:** Cecilia P Chung, S Todd Callahan, William O Cooper, Katherine T Murray, Kathi Hall, Judith A Dudley, C Michael Stein, Wayne A Ray

**Affiliations:** Division of Rheumatology, Department of Medicine, Vanderbilt University School of Medicine, T-3113 MCN, 1161 21st Ave. S., Nashville, TN 37232 USA; Department of Health Policy, Vanderbilt University School of Medicine, Nashville, TN 37232 USA; Department of Pediatrics, Vanderbilt University School of Medicine, Nashville, TN 37232 USA

**Keywords:** Opioid, Poisoning, Toxicity, Children, Validation, Algorithm

## Abstract

**Background:**

The use of opioids is increasing in children; therefore, opioid toxicity could be a public health problem in this vulnerable population. However, we are not aware of a published algorithm to identify cases of opioid toxicity in children using administrative databases. We sought to develop an algorithm to identify them. After review of literature and de-identified computer profiles, a broad set of ICD-9 CM codes consistent with serious opioid toxicity was selected. Based on these codes, we identified 195 potential cases of opioid toxicity in children enrolled in Tennessee Medicaid. Medical records were independently reviewed by two physicians; episodes considered equivocal were reviewed by an adjudication committee. Cases were adjudicated as Group 1 (definite/probable), Group 2 (possible), or Group 3 (excluded).

**Results:**

Of the 195 potential cases, 168 (86.2%) had complete records for review and 85 were confirmed cases. The overall positive predictive value (PPV) for all codes was 50.6%. The PPV for codes indicating: unintentional opioid overdose (25/31) was 80.7%; intentional opioid overdose (15/30) was 50.0%, adverse events (33/58) was 56.9%, the presence of signs or symptoms compatible with opioid toxicity (12/47) was 25.5%, and no cases were confirmed in records from the two deaths. Of the confirmed cases, 65.8% were related to therapeutic opioid use.

**Conclusion:**

For studies utilizing administrative claims to quantify incidence of opioid toxicity in children, our findings suggest that use of a broad set of screening codes coupled with medical record review is important to increase the completeness of case ascertainment.

## Background

There is a well-recognized epidemic of hospitalizations and deaths related to the increasing use of opioid analgesics in adults [1–3]. From 1999 to 2008 in the United States, rates of opioid sales, toxicity, and death related to opioid use tripled, and recent data indicate that opioid toxicity accounts for 73.8% of all deaths attributed to a prescription drug [4].

Although there are fewer studies in children, this vulnerable population also appears to be at high risk for opioid toxicity. A study of 960 randomly selected medical records from 12 children’s hospitals identified 107 adverse drug events with more than half attributable to opioid analgesics [5]. Deaths have been reported in young children related to therapeutic use of codeine [6] and hydrocodone [7] in doses within or moderately exceeding recommended pediatric limits.

Despite their potential for serious adverse events, opioids are increasingly prescribed for adolescents. Opioid prescriptions for patients between 15 and 19 years of age doubled from 1994 to 2007, with estimates that opioids are prescribed in nearly 6% of ambulatory and emergency department visits made by adolescents in the US [8].

Given the large number of pediatric patients receiving prescribed opioids, there is an urgent need for fundamental epidemiologic studies to inform the risk–benefit decisions of prescribers and families. An essential component of these epidemiologic studies is the identification of serious adverse reactions related to opioids. Epidemiologic studies in adults have developed procedures to identify hospitalizations and deaths related to opioid use [3, 9]. However, we are unaware of similar studies in children.

Studies specifically aiming to evaluate children are important because there are significant differences between children and adults. For example, during growth and development, changes in drug metabolizing enzyme activity result in age-related differences in drug disposition [10]. Moreover, focus on only deaths and hospitalizations is likely to underestimate the occurrence of serious adverse events in pediatric practice, given the low frequency of these events in children. Thus, the primary purpose of the present study was to develop and test procedural tools for identification of opioid toxicity in children for a broad spectrum of adverse events, including visits to emergency departments, hospital admissions, and deaths. The secondary purpose was to assess the positive predictive value (PPV) of specific groups of diagnostic codes.

We designed our procedures for large administrative databases, given their recognized utility for pharmacoepidemiology [11]. This process included the identification of medical encounters in children likely to indicate serious opioid toxicity, procedures for adjudication of cases of possible opioid toxicity, and acquiring preliminary data on the PPV of the screening procedures as well as the circumstances of the opioid use.

## Methods

### Setting and study population

#### Tennessee Medicaid

The study was conducted in children 2–17 years of age enrolled in the Tennessee Medicaid program between 1999 and 2011. This setting was chosen because Tennessee Medicaid included large numbers of children (49.7% of all Tennessee Medicaid enrollees) and the Medicaid files permitted efficient identification of children who filled a prescription for an opioid, potential adverse opioid effects, and pertinent demographic and clinical covariates [11]. Study files included enrollment, pharmacy (filled prescription), outpatient and inpatient visits, as well as linked state hospital discharge (with emergency department visits) and death certificate files [11].

#### Cohort and follow-up

We sought to reduce the confounding by symptoms related to terminal illness and serious medical disorders. Therefore, we excluded children with prior medical care encounters indicating serious illnesses in the previous 12 months (including cancer, sickle cell anemia, congenital anomalies, etc.), history of organ transplant, institutional residence, and/or history of drug abuse (Table 5 in “Appendix”). For each child in the cohort and for each opioid prescription filled by that child, study follow-up included the period between the filling of the prescription and 14 days following the end of the prescribed days of supply. Although opioid toxicity most commonly occurs during current use of the drug, adverse events may delayed, or, given that opioids are often prescribed to be taken “as needed”, the period of actual use may extend beyond the dispensed days of supply.

#### Development study sample

Our ultimate goal is to study the epidemiology of serious opioid toxicity in a large cohort of children prescribed opioids. Our strategy for identifying serious opioid toxicity was to first identify potential cases from medical care encounters and then to confirm these by adjudication of medical records. The purpose of the current development study was to test and refine the procedures for identifying opioid-related adverse events. The development study was restricted to encounters at five major centers with a high volume of Tennessee Medicaid pediatric patients for which we had access to medical records.

### Medical encounters indicating opioid toxicity

#### Clinical spectrum of opioid toxicity

Opioids have a wide range of adverse effects [12]. The most serious is overdose, nearly always accompanied by respiratory or central nervous system (CNS) depression [13–15]. Other adverse effects include milder CNS effects, constipation, nausea, vomiting, dermatologic reactions, urinary retention, myoclonus, seizures (with meperidine) and metabolic disorders [12, 14, 16].

#### Encounters indicating potential cases of opioid toxicity

Serious opioid toxicity was defined as Group 1: definite/probable, or Group 2: possible based on medical record review. Encounters of interest were restricted to emergency department visits, hospital admissions, and deaths. Potential cases were identified from ICD-9-CM diagnostic codes, either primary or secondary, recorded for the encounter.

There were four groups of codes. First, were the external cause of injury codes that indicate medication poisoning/overdose (Table 6 in “Appendix”). Because all cohort follow-up consisted of either current or very recent use of an opioid, we hypothesized there was a considerable prior probability of opioid involvement in an adverse drug reaction. Thus, in this development study, we included codes that indicated an opioid or unspecified analgesic (codes in Group A of tables) as well as those that indicated an unspecified medication (codes in Group C of the tables), unless the encounter also indicated a specific drug that was not an opioid (codes in Group B). We did not consider encounters with other specified medications, because, given that the reaction had been attributed to another drug (e.g., hypoglycemia related to insulin), we thought it would be unlikely to be adjudicated as due to an opioid. Second, were the external cause of injury codes indicating an adverse effect in therapeutically appropriate use (Table 7 in “Appendix”). Third, were diagnosis codes indicating symptoms of the most serious opioid toxicity: respiratory depression and adverse CNS effects (Table 8 in “Appendix”), even in the absence of an external cause of injury code. Finally, records for all deaths during follow-up were reviewed, regardless of the cause of death diagnosis codes.

There were several other groups of codes initially considered but ultimately not included in those for identifying potential cases. These included diagnoses indicating symptoms possibly due to less serious toxicity (such as rash, nausea, or constipation) in the absence of a code indicating an adverse drug effect. These were excluded because of the large number of such encounters and the difficulty of attributing them to an opioid even with medical record review. We also excluded some less specific codes for respiratory and CNS symptoms (e.g., dizziness), based on their relative commonness, difficulty of attribution to an opioid, analysis of computer profiles, and preliminary analysis of the development study data. We initially considered symptoms related to metabolic disorders, but preliminary analysis suggested these were infrequent and had poor PPV. We also considered administration of naloxone as an indicator of overdose, but a preliminary review indicated that naloxone was most frequently used to reverse the effects of opioid administration following a surgical procedure. However, naloxone use did not trigger exclusion from the cohort.

### Adjudication procedures

**Figure 1:**
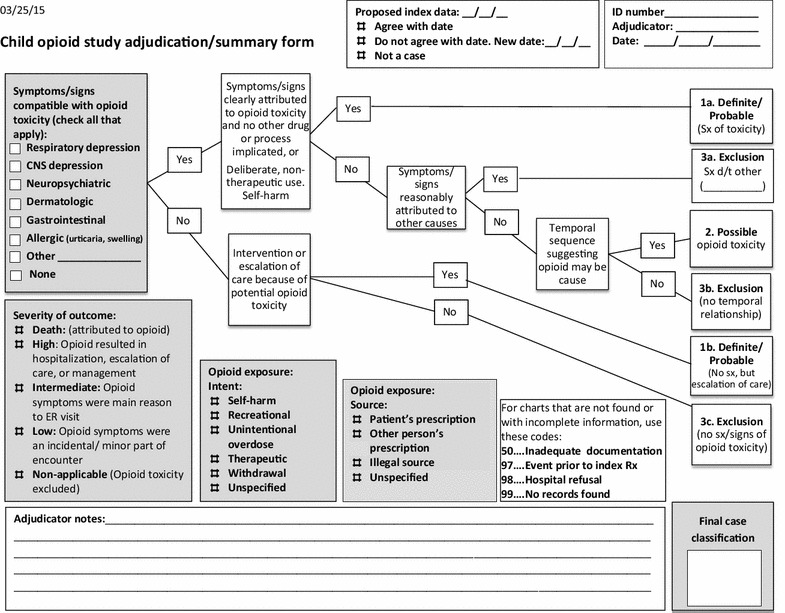
Child opioid study adjudication/summary form.

After de-identification, all medical records were independently reviewed and adjudicated by two study physicians (STC and CPC) using the study instrument (Figure 1 in “Appendix”). Episodes for which there was disagreement among adjudicators were subsequently adjudicated by all the investigators. Following standard procedures, we reviewed complete medical records for evidence of symptoms and signs clearly attributed to opioid toxicity with no other process implicated. Other clinical information included temporal sequence of events, medical interventions, escalation of care and evidence of symptoms or signs attributable to other causes. Consensus was reached after discussion of all available evidence.

Adjudicated episodes were classified into one of the following mutually exclusive categories:

*Group 1: Definite/Probable opioid toxicity* There were two scenarios:

1. The presence of both of the following criteria: (1) signs and symptoms compatible with opioid toxicity and attributed by the treating physician as related to an opioid poisoning, overdose or adverse event; and (2) no other drug or disease process implicated in the presence of these signs or symptoms.
2. Direct intervention or escalation of care because of potential opioid toxicity (for example: admission to the Intensive Care Unit after inadvertent overdose of hydrocodone).

*Group 2: Possible opioid toxicity* These episodes met both of the following criteria: (1) signs or symptoms compatible with opioid toxicity that were not clearly attributed directly to opioid toxicity but not attributed to other causes; and (2) the temporal sequence suggested that opioid use was a possible cause of signs or symptoms.

*Group 3: excluded, opioid toxicity unlikely* There were three scenarios in which opioid toxicity was excluded:

1. Presence of signs or symptoms compatible with opioid toxicity but attributed to other causes (for example: vomiting attributed to gastrointestinal infection by treating physician).
2. Presence of signs and/or symptoms compatible with, but not clearly attributed to opioid toxicity (but not attributed to other causes either); and lack of a temporal sequence suggesting that opioids were a possible cause of signs or symptoms.
3. Neither signs nor symptoms compatible with opioid toxicity nor escalation of care because of potential opioid toxicity.

Our definition did not consider findings from urine and serum drug screens for opioids. These were not consistently performed and have been shown to have limited utility in the diagnosis of acute opioid toxicity [12].

#### Severity of the outcome

Cases that met the criteria for probable or possible opioid toxicity were classified according to severity of the event:

- Death: attributed to opioid.
- High: the adverse effect resulted in hospitalization or escalation of care or management.
- Intermediate: the opioid-related symptoms were the primary reason for an emergency room visit.
- Low: the opioid-related symptoms were an incidental or minor part of the encounter.

#### Opioid exposure

For each probable or possible case, we also recorded both the circumstances of the opioid exposure as well as the source of the opioid. The circumstances of exposure included: (1) self-harm—any indication of attempted suicide or intentional overdose, (2) recreational ingestion, (3) unintentional overdose—e.g., parent inadvertently administered an extra dose, (4) therapeutic use—the medication was taken as prescribed, (5) opioid withdrawal, (6) unspecified—no indication of circumstances or intent documented. The source of the opioid related to the adverse event was: (1) the patient’s prescription, (2) another person’s prescription, (3) an illegal source, or (4) not specified.

### Statistical analyses

Statistical analyses were conducted in two steps. First, tables were constructed specifying whether clinical criteria for opioid toxicity (definitive, probable and possible) were met according to review of medical records and PPVs were calculated. Secondary analyses included calculation of PPV according to time elapsed since the end of the days of supply (through 7 days and 8–14 days).

Cases adjudicated as Group 1 (definite/probable) or Group 2 (possible) opioid toxicities were further classified by severity and by exposure, as detailed above. Second, based on final adjudication, positive predictive values were calculated. Reliability was expressed as kappa statistics, excluding 12 initial cases used to refine the adjudication procedures. Analyses were conducted with the use of STATA 12 (StataCorp. 2011. Stata Statistical Software: Release 12, College Station, TX: StataCorp LP).

The study was approved by the Institutional Review Board at Vanderbilt University and the Tennessee Medicaid program.

## Results

### Demographic of study population

Based on the screening codes (listed in the “Appendix”), 195 potential cases of opioid toxicity in children from five hospitals in Tennessee were identified. These children were 11.3 ± 5.0 years old on average and predominantly Caucasian (59%) and female (57%).

### Positive predictive value

From the 195 potential cases, records for four cases (2%) did not have enough information to adjudicate and 23 (12%) were not found. Of the 168 remaining potential cases, 85 were adjudicated as Group 1 (definite/probable) or Group 2 (possible) opioid toxicity event (PPV = 50.6%) (Table 1).

**Table 1:** Positive predictive value for codes identifying potential opioid toxicity in children

	Total	Confirmed^a^	
		n	PPV (%)
Overall	168	85	50.6
Stratified analyses by type of diagnosis			
Opioid overdose, intentional	30	15	50.0
Opioid overdose, unintentional	31	25	80.7
Potential opioid adverse effect	58	33	56.9
Symptoms indicative of opioid overdose (CNS, respiratory)	47	12	25.5
Death, any cause	2	0	0

*PPV* positive predictive value.

^a^Confirmed = Group 1 (definitive/probable) or Group 2 (possible).

We calculated PPVs for specific diagnostic codes. Of 31 potential cases identified as unintentional poisoning/overdose, 25 were confirmed (PPV = 80.7%). Thirty-three of the 58 cases identified based on codes for adverse effect of drugs in therapeutic use were confirmed, with a PPV = 56.9%. Lower PPVs were found for patients with codes indicating intentional overdose (PPV = 50.0%), or symptoms compatible with opioid toxicity (25.5%). Although the numbers were small, cases identified on the basis of CNS symptoms had a PPV = 28.1%. The PPV for cases identified based on respiratory symptoms was lower (20.0%) (Table 2). There were two deaths, but after review of records the causes of death in both cases were not compatible with opioid toxicity.

**Table 2:** Utility of symptom screening codes

	n	n, confirmed	PPV (%)
All symptoms	47	12	25.5
CNS	32	9	28.1
Respiratory	15	3	20.0

We calculated the PPVs according to the time elapsed since the end of the days of supply (Table 3). There were 121 cases identified between the filling of the prescription and 7 days following the end of the days of supply, of which 69 were confirmed, with a PPV = 57.0%. Of the potential 47 potential cases identified 8–14 days after the end of the days of supply, 16 were confirmed (PPV = 34.0%).

**Table 3:** Positive predictive values for screening codes according to time since end of days of supply

	n	n, confirmed	PPV (%)
Entire sample	168	85	50.6
Date of prescription fill through 7 days following end of days of supply	121	69	57.0
Days 8–14 following end of days of supply	47	16	34.0

### Severity of outcome, intent, and source of the opioid

With regard to the severity of the outcomes, 27 (31.8%) cases were classified as high, 52 (61.2%) as intermediate, and six (7.1%) as low severity. Based on intent of opioid use, 56 (65.8%) cases were related to therapeutic use, 10 (11.8%) to self-harm, nine (10.6%) to unintentional overdose, and four (4.7%) to recreational use of opioids. There was one case with records indicating opioid withdrawal and in six cases (7.1%) it was not possible to determine the intent. We also assessed the source of opioid. The majority of the cases (n = 76, 89.4%) had records supporting the use of a prescribed opioid, while the use of another person’s prescription was noted in only four cases. There were no cases with indication of an illegal source and in six cases (7.1%) it was not possible to specify the source (Table 4).

**Table 4:** Severity of outcome, intent of opioid use, and source of the opioid

	n	%
Severity of event		
High	27	31.8
Intermediate	52	61.2
Low	6	7.1
Intent		
Therapeutic use	56	65.8
Self-harm	10	11.8
Unintentional overdose	9	10.6
Other	11	13.0
Source of opioid		
Patient’s prescription	76	89.4
Other person’s prescription	4	4.7
Other	6	7.1

### Inter-rater agreement

All cases were initially reviewed by two clinicians. From the 156 cases available for estimation of inter-rater reliability, 13 cases were brought to the adjudication committee for further discussion. There was agreement in 91.7% (the expected agreement was 50.6%) and the kappa statistics was 0.83 (p < 0.001). In eight of these 13 cases, one reviewer initially adjudicated the case as Group 2 (possible) and the other as Group 3 (exclusion).

## Discussion

To the best of our knowledge, this is the first study describing the development and validation of a procedural tool, based on identification of potential cases with ICD codes and adjudication of individual cases with review of medical records, to identify opioid toxicity in children for use in pharmacoepidemiologic studies in automated databases. Our analyses showed that the study codes for identifying adverse opioid effects had a low PPV, which suggests that review of medical records is important.

Many symptoms of opioid toxicity have a broad differential diagnosis and may be related to other concurrent factors, including other medications [17]. Furthermore, it is well recognized that medications affect children and adult differently. Although others have developed procedures to identify fatal cases of opioid overdose from diagnosis codes [3], these results are not necessarily applicable to non-fatal outcomes. Furthermore, we cannot assume that data from adults are necessarily applicable to children.

Thus, we identified a set of screening codes for opioid toxicity specifically for a pediatric population. To adjudicate potential cases, we followed a detailed procedure that began with confirming the presence of symptoms or signs compatible with opioid toxicity. We excluded cases in which the treating physician attributed the symptoms and signs to other causes. The temporal sequence of events and intervention/escalation of care were also considered. The initial complete review of each medical record and its adjudication were done by two experienced physicians, blinded to the other’s adjudication. Although the interrater reliability kappa of 0.84 indicates substantial agreement [18]; there were 7.7% cases in which the reviewers disagreed. Thus, our adjudication process should improve accuracy relative to review and adjudication by a single investigator.

Although the overall PPV was low, PPVs differed greatly according to the type of diagnosis. Cases that were identified based on codes indicating opioid or analgesic unintentional overdose/poisoning had the highest PPV; but they only accounted for 25 of the 85 (29.4%) confirmed cases. This suggests that studies to quantify the incidence of opioid adverse effects should not be limited to these codes. In contrast, cases identified based on diagnosis codes consistent with symptoms of opioid toxicity, but with no mention of a drug, had a PPV of only 25.5%. However, such cases accounted for 12 (14.1%) of the confirmed cases. Therefore, failure to use symptom-related codes will lead to underestimation of the incidence of opioid toxicity.

Several limitations should be considered. First, determination of causality was based primarily on the judgment of the treating physician, as documented in the medical records. This limitation, inherent to retrospective studies, may be particularly important for less serious opioid adverse effects with multiple causes, such as dermatologic or gastrointestinal reactions. Second, the algorithm was developed to identify cases of opioid toxicity related to hospitalization or an emergency department visit; thus, less serious events were not captured. Third, because the present study is restricted to children with a filled opioid prescription, our procedures are designed for study of therapeutic use of opioids. Fourth, 11.8% of records, primarily from early years, were unavailable for review, most commonly because the records were already destroyed. Fifth, we do not include data on treatment duration. Finally, the study was conducted in children enrolled in Tennessee Medicaid, and because our primary area of concern was relatively healthy children receiving opioids therapeutically we exclude children with serious conditions, which may limit generalizability to other databases and clinical settings.

## Conclusion

In summary, the overall positive predictive value of diagnosis codes indicating opioid toxicity was low. Our findings suggest that for incidence studies, a two-step process would allow inclusion of a broader range of screening diagnostic codes and thus would capture a larger fraction of cases of opioid toxicity in the study population.

## Appendix

See Figure 1 and Tables 5, 6, 7 and 8.

**Table 5:** Serious illnesses

Disease	Definition
Cancer	Diagnosis of cancer (except for non-melanoma skin cancers) Selected antineoplastic agents
Hematologic diseases	Sickle cell diagnosis, aplastic anemia
Neuromuscular diseases	Cerebral palsy, muscular dystrophy, multiple sclerosis, ALS, quadriplegia, paraplegia, hemiplegia, spinal cord injury, stroke
Chromosomal anomalies	Down’s syndrome, trisomy 13, trisomy 18, autosomal deletion syndrome and others
Other congenital anomalies-non cardiovascular	Cystic fibrosis, anencephalus, spina bifida, hydrocephalus, microcephalus, encephalocele
Congenital anomalies-cardiovascular	Common truncus, transposition great vessels, tetralogy of Fallot, common ventricle, endocardial cushion defect, pulmonary atresia, tricuspid atresia, hypoplastic left heart, coarctation of aorta, other anomalies of aorta, total anomalous pulmonary venous connection
Gastrointestinal diseases	Ulcerative colitis, Crohn’s disease Liver disease: acute and subacute necrosis of the liver, chronic liver disease and cirrhosis, hepatic encephalopathy, portal hypertension, hepatorenal syndrome, other sequelae of chronic liver disease, other disorders Hospitalization for any other liver disease: abscess of the liver, portal pyemia, hepatopulmonary syndrome, other specified disorders of the liver Acute or chronic pancreatitis
Mental retardation	Moderate to profound intellectual disability
HIV and other serious infections	Diagnosis of HIV Use of antiretroviral agents appropriate for HIV or pentamidine Hepatitis B and/or C Treatment for viral hepatitis Tuberculosis Treatment for tuberculosis
Other immunologic conditions	Immune deficiencies (deficiency of humoral immunity, deficiency of cell-mediated immunity, combined immunity deficiency, unspecified immunity deficiency)
Renal disease	Diagnosis or procedure code for dialysis or end-stage renal disease outside of the hospital
Cardio-respiratory diseases	Any diagnosis of primary pulmonary hypertension Inpatient diagnosis of chronic respiratory failure, cardio-respiratory failure, or pulmonary heart disease Tracheostomy (excluding temporary)
Organ transplant	Includes kidney, heart, lung, liver, bone marrow, and pancreas Medications: azathioprine, cyclosporine, tacrolimus (except derm preparation), mycophenolate mofetil, mycophenolate sodium, sirolimus, daclizumab, antithymocyte immune, bevacizumab, basiliximab, muromonab
Other serious illness	Hospice care Diagnosis of coma, vegetative state, debility, cachexia Total parenteral nutrition/gastrostomy Gangrene Intravenous medications outside of the hospital Severe metabolic disorders: disturbances of the amino-acid transport, disturbances of carbohydrate, mucopolysaccharidosis, other specified disorders of the metabolism

**Table 6:** ICD9-CM diagnosis codes indicating potential medication overdose

	Code	Diagnosis
A. Opioid or unspecified analgesic		
1.	965.0	Poisoning by analgesics, antipyretics, and antirheumatics, opiates and related narcotics
2.	965.8	Other specified analgesics and antipyretics, pentazocine
3.	965.9	Unspecified analgesic and antipyretic
4.	E850.0	Accidental poisoning by analgesics, antipyretics, and antirheumatics, heroin
5.	E850.1	Accidental poisoning by analgesics, antipyretics, and antirheumatics, methadone
6.	E850.2	Accidental poisoning by analgesics, antipyretics, and antirheumatics, other opiates and related narcotics
7.	E850.8	Accidental poisoning by analgesics, antipyretics, and antirheumatics, pentazocine
8.	E850.9	Accidental poisoning by analgesics, antipyretics, and antirheumatics, unspecified analgesic or antipyretic
9.	E950.0*	Self-inflicted injury, analgesics, antipyretics, and antirheumatics
10.	E980.0*	Injury undetermined whether accidentally or purposely inflicted, analgesics, antipyretics, and antirheumatics
B. Other specified medication. Code only qualifies if not otherwise listed (in A, C)		
11.	960–979	
12.	E850–E858	
13.	E950.0–E950.4*	
14.	E980.0–E980.4*	
C. Unspecified medication or substance		
15.	292.1	Drug-induced psychotic disorders
16.	292.2	Pathological drug intoxication
17.	292.8	Other specified drug-induced mental disorders
18.	292.9	Unspecified drug-induced mental disorder
19.	909.0	Late effect of poisoning due to drug, medicinal or biological substance
20.	977.9	Poisoning by other and unspecified drugs and medicinal substances
21.	E858.9	Accidental poisoning by other drugs, unspecified drug
22.	E950.5*	Self-inflicted injury, unspecified drug or medicinal substance
23.	E980.5*	Injury undetermined whether accidentally or purposely inflicted, unspecified drug or medicinal substance
24.	E950.9*	Self-inflicted injury, other and unspecified solid and liquid substances
25.	E980.9*	Injury undetermined whether accidentally or purposely inflicted, other and unspecified solid and liquid substances

Codes with an * denote possible intentional injuries. Unlike Dunn and colleagues [3], we do not include codes for intentional injuries where these specify a means other than drug (e.g. firearm). We also do not include the injury codes for unspecified means; preliminary review established these were nearly all E988.9, and had little probability of relation with an endpoint.

**Table 7:** ICD9-CM diagnosis codes indicating potential adverse effects for medications used in a therapeutically appropriate manner

	Code	Diagnosis
A. Opioid or unspecified analgesic		
1.	E935.0	Analgesics, antipyretics, and antirheumatics, heroin
2.	E935.1	Analgesics, antipyretics, and antirheumatics, methadone
3.	E935.2	Analgesics, antipyretics, and antirheumatics, other opiates and related narcotics
4.	E935.8	Analgesics, antipyretics, and antirheumatics, pentazocine
5.	E935.9	Analgesics, antipyretics, and antirheumatics, unspecified analgesic or antipyretic
B. Other specified medication. For ranges, code only qualifies if not otherwise listed (in A, C)		
6.	E930–E947.9	
C. Unspecified medication		
7.	693.0	Dermatitis due to substances taken internally, drugs and medications
8.	909.5	Late effect of adverse effect of drug, medicinal or biological substance
9.	995.2	Other and unspecified adverse effect of drug, medicinal and biological substance (due) to correct medicinal substance properly administered
10.	E947.9	Unspecified drug or medicinal substance

We exclude dermatitis (692.3, topical) as no topical drugs are included in the study.

**Table 8:** ICD9-CM diagnosis codes for opioid respiratory or CNS symptoms

	Code	Diagnosis
A. Respiratory depression^a^ in the absence of known causes^b^		
1.	518.0	Pulmonary collapse
2.	518.81	Acute respiratory failure
3.	518.82	Other pulmonary insufficiency, not elsewhere classified
4.	518.84	Acute and chronic respiratory failure
B. Central nervous system^c^		
5.	780.0	Alteration of consciousness
6.	780.01	Coma
7.	780.02	Transient alteration of awareness
8.	780.09	Other alteration of consciousness
9.	780.1	Hallucinations
10.	780.97	Altered mental state
11.	781.3	Lack of coordination

^a^Does not include pneumonia (486), chronic airway obstruction (496), shortness of breath (786.05,786.09), or painful respiration (786.52). Based on the preliminary analysis, also does not include symptoms: 786.00 respiratory abnormality, unspecified, 786.03 apnea, 799.0 asphyxia and hypoxemia, 799.01 asphyxia, 799.02 hypoxemia, 799.1 respiratory arrest. Pilot indicated very low PPV for these latter codes.

^b^For the day the prescription was filled or the preceding 6 days, no encounter with a diagnosis of pneumonia or other respiratory infections, pleural effusion, pneumothorax; or a procedure for thoracentesis or thoracostomy.

^c^Does not include dizziness (780.4) or abnormal gait (781.2).

## Abbreviations

CNS: central nervous system
ICD: International Classification of Diseases
PPV: positive predictive value

## References

[1] Okie S (2010). A flood of opioids, a rising tide of deaths. N Engl J Med.

[2] Kuehn BM (2007). Opioid prescriptions soar: increase in legitimate use as well as abuse. JAMA.

[3] Dunn KM, Saunders KW, Rutter CM, Banta-Green CJ, Merrill JO, Sullivan MD (2010). Opioid prescriptions for chronic pain and overdose. Ann Intern Med.

[4] Centers for Disease Control and Prevention (2011) Vital signs: overdoses of prescription opioid pain relievers—United States, 1999–2008. MMWR Morb Mortal Wkly Rep 60:1487–1492 22048730

[5] Takata GS, Mason W, Taketomo C, Logsdon T, Sharek PJ (2008). Development, testing, and findings of a pediatric-focused trigger tool to identify medication-related harm in US children’s hospitals. Pediatrics.

[6] Ferreirós N, Dresen S, Hermanns-Clausen M, Auwaerter V, Thierauf A, Müller C (2009). Fatal and severe codeine intoxication in 3-year-old twins—interpretation of drug and metabolite concentrations. Int J Legal Med.

[7] Madadi P, Hildebrandt D, Gong IY, Schwarz UI, Ciszkowski C, Ross CJ (2010). Fatal hydrocodone overdose in a child: pharmacogenetics and drug interactions. Pediatrics.

[8] Fortuna RJ, Robbins BW, Caiola E, Joynt M, Halterman JS (2010). Prescribing of controlled medications to adolescents and young adults in the United States. Pediatrics.

[9] Landen MG, Castle S, Nolte KB, Gonzales M, Escobedo LG, Chatterjee BF (2003). Methodological issues in the surveillance of poisoning, illicit drug overdose, and heroin overdose deaths in new Mexico. Am J Epidemiol.

[10] de Wildt SN, Tibboel D, Leeder JS (2014). Drug metabolism for the paediatrician. Arch Dis Child.

[11] Ray WA, Griffin MR (1989). Use of Medicaid data for pharmacoepidemiology. Am J Epidemiol.

[12] Boyer EW (2012). Management of opioid analgesic overdose. N Engl J Med.

[13] Hayes BD, Klein-Schwartz W, Doyon S (2008). Toxicity of buprenorphine overdoses in children. Pediatrics.

[14] Sachdeva DK, Stadnyk JM (2005). Are one or two dangerous? Opioid exposure in toddlers. J Emerg Med.

[15] Boyer EW, McCance-Katz EF, Marcus S (2010). Methadone and buprenorphine toxicity in children. Am J Addict.

[16] Kyff JV, Rice TL (1990). Meperidine-associated seizures in a child. Clin Pharm.

[17] Kramer MS (1981). Difficulties in assessing the adverse effects of drugs. Br J Clin Pharmacol.

[18] Viera AJ, Garrett JM (2005). Understanding interobserver agreement: the kappa statistic. Fam Med.

